# A Disseminated Mycobacterium Abscessus Infection in a Patient Affected by Pulmonary Graft versus Host Disease: Case Report with a Revision of Literature

**DOI:** 10.3390/jcm11092410

**Published:** 2022-04-25

**Authors:** Andrea Bernardelli, Alberto Schena, Alessia Savoldi, Chiara Colato, Valentina Baretta, Emiliano D’Alessandro, Giulia Zamboni, Mehrdad Shoushtari Zadeh Naseri, Flavio Favaro, Marta Peracchi, Donatella Schena, Angelo Andreini, Simone Cesaro, Cristina Tecchio

**Affiliations:** 1 Section of Hematology and Bone Marrow Transplant Unit, Department of Medicine, Verona University, 37134 Verona, Italy; andrea.bernardelli@studenti.univr.it (A.B.); alberto.schena@studenti.univr.it (A.S.); valentina.baretta@studenti.univr.it (V.B.); angelo.andreini@aovr.veneto.it (A.A.); 2 Infectious Diseases Unit, Department of Diagnostics and Public Health, Verona University, 37134 Verona, Italy; alessia.savoldi@univr.it; 3 Section of Pathology, Verona University, 37134 Verona, Italy; chiara.colato@aovr.veneto.it (C.C.); emiliano.dalessandro@aovr.veneto.it (E.D.); 4 Department of Radiology, Verona University, 37134 Verona, Italy; giulia.zamboni@aovr.veneto.it; 5 Department of Nuclear Medicine, Verona University, 37134 Verona, Italy; naseri.mehrdad@gmail.com; 6 Section of Microbiology, Department of Diagnostics and Public Health, Verona University, 37134 Verona, Italy; flavio.favaro@aovr.veneto.it; 7 Microbiology and Virology Unit, Padua University, 35122 Padova, Italy; marta.peracchi@aopd.veneto.it; 8 Section of Dermatology and Venereology, Department of Medicine, Verona University, 37134 Verona, Italy; donatella.schena@aovr.veneto.it; 9 Pediatric Hematology Oncology, Verona University Hospital, 37134 Verona, Italy; simone.cesaro@aovr.veneto.it

**Keywords:** *Mycobacterium abscessus complex*, nontuberculous mycobacteria, haploidentical hematopoietic stem cell transplant, pulmonary chronic graft versus host disease

## Abstract

*Mycobacterium abscessus* *complex*, hereinafter *Mab*, is a taxonomic group of rapidly growing, nontuberculous mycobacteria (NTM). Despite major advances in understanding virulence, pathogenicity and mechanism of antibiotic resistance, *Mab* remains a significant cause of pulmonary and extra-pulmonary disease. Herein, we describe a disseminated, macrolide-resistant, *Mab* subspecies *abscessus* infection occurring in a severely immune-compromised 34-year-old allotransplanted female patient affected by pulmonary chronic graft versus host disease (cGVHD). The infection was characterized by hematogenous spread, and besides lungs, it involved skin, and soft tissues, resulting in a highly debilitating, painful, and finally fatal disease. Our case describes the severe impact of *Mab* infections in the setting of allogeneic hematopoietic stem cells transplant (alloHSCT) and related complications. It also highlights the unmet need of preventive and surveillance measures together with the urgency of developing effective vaccines and drugs against emerging NTM. The scarce literature regarding *Mab* infections in alloHSCT patients is also reviewed.

## 1. Introduction

NTM comprise a group of more than 190 species of *Mycobacterium* other than *Mycobacterium tuberculosis* and *Mycobacterium leprae* [1]. Based on growth characteristics NTM can be further distinguished into rapidly-growing (e.g., *Mycobacterium fortuitum, Mycobacterium chelonae,* and *Mycobacterium abscessus*) and slow-growing (e.g., *Mycobacterium avium, Mycobacterium kansasii*, and *Mycobacterium marinum*) species [2]. Importantly, the last decades have witnessed a global increase of NTM infections, that in turn have been favored by host-related factors such as population aging, lung diseases, broad-spectrum antibiotic therapy, and immune deficiencies [3,4]. While lung disease is the most frequent manifestation, subjects with innate or acquired immune impairment are also at risk for disseminated NTM infections [3]. Patients undergoing alloHSCT are severely immunosuppressed due to either the length of the immune reconstitution process or the immunosuppressive treatments administered for GVHD prophylaxis and/or cure. Scattered information is currently available about characteristics and impact of NTM infections in alloHSCT setting, with two large retrospective studies on adult patients mostly reporting isolated lung involvement [5,6].

*Mab* is a rapidly growing mycobacterium species composed of the subspecies (subsp) *abscessus*, *bolletii*, and *massiliense* [4]. Several names have been applied to these three organisms throughout years, thus leading to some inconsistencies in the medical literature [7]. In recent years, *Mab* has become an emerging cause of severe pulmonary disease, especially in patients with cystic fibrosis or underlying structural lung damage such as bronchiectasis [3,8,9]. Nonetheless, bloodstream, skin and mucosal infections have been also described [4]. Importantly, *Mab* is regarded as one of the most difficult-to-treat NTM due to its multiple drug-resistance mechanisms [7,10].

Herein, we describe a disseminated *Mab* subsp *abscessus* infection occurring in a severely immune-compromised 34-year-old allotransplanted female patient affected by pulmonary cGVHD, a donor-derived immune-mediated disease causing structural lung damages and requiring long-term immune suppressive treatments [11]. In our patient the infection was characterized by hematogenous spread, and besides lungs, it involved skin, and soft tissues. Despite multi-antibiotic therapy, the patient died within 4.5 months since diagnosis, never achieving microbiological clearance and displaying macrolide resistance. This case describes the potential severe impact of *Mab* infections in alloHSCT patients, highlighting the need to develop effective preventive measures and therapies. A revision of the scarce literature on *Mab* infections in alloHSCT patients is also presented.

## 2. Case Report

**Figure 1:**
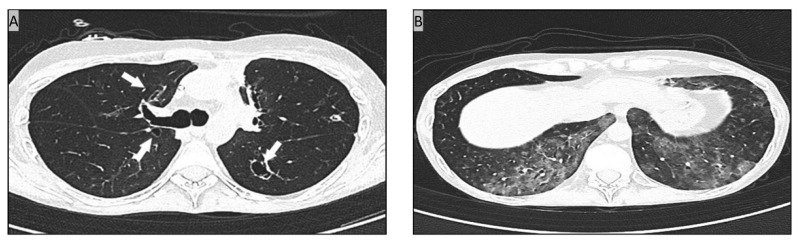
Chest HRCT showing pulmonary cavitated lesions with thick walls (arrows) and nodules in the upper lobes (**A**), and extensive ground-glass opacities in the lower lobes (**B**).

**Figure 2:**
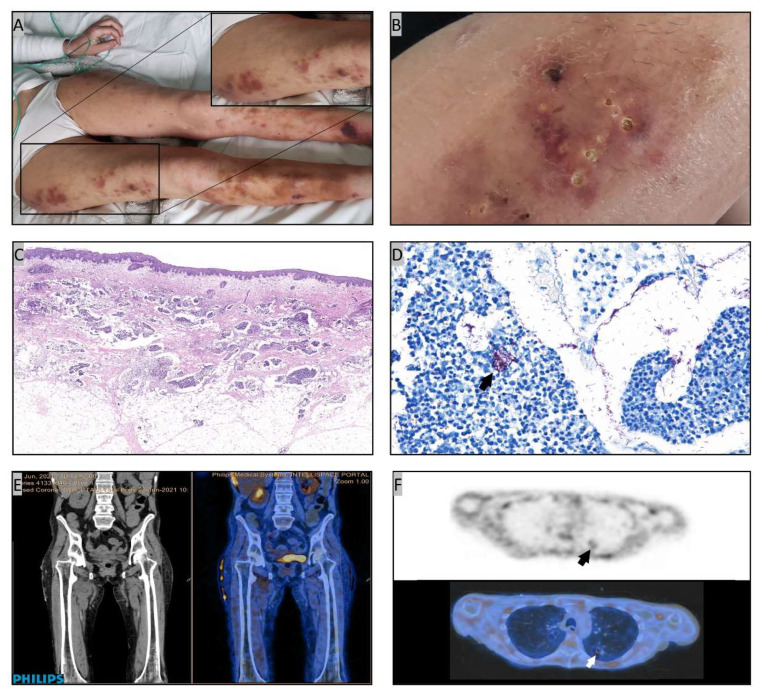
Overview of leg skin lesions with a detail of nodules on the right thigh (**A**), and photographic enlargement of abscessed lesions (**B**); histological analysis of skin biopsy evidencing a diffuse nodular suppurative inflammation with pseudocystic spaces involving the dermis and subcutis (Hematoxylin-eosin, original magnification ×8) (**C**), and abundant microorganisms (arrow) identifiable within the pseudocystic spaces by Ziehl-Neelsen staining (original magnification ×400) (**D**); FDG-CT/PET images showing an increased uptake at skin and soft tissues levels, with those of the right tight and sacrum presenting the maximum value (SUV 5.4) (**E**), and an increased uptake (arrow) in correspondence of lung lesions (**F**).

Our patient underwent haploidentical hematopoietic stem cell transplantation (haploHSCT) in June 2016, due to a grey-zone lymphoma—stage IIB bulky mediastinal—that was in partial response after 4 lines of therapy including autologous HSCT. Because of residual disease persistence at CT/PET, haploHSCT was followed by 5 donor lymphocyte infusions (DLIs) that allowed the achievement of disease remission. The diagnosis of pulmonary cGVHD was made in October 2017, 6 months after DLI completion, on the basis of newly developed respiratory symptoms, pulmonary function alterations (FEV1 < 70%, FEV1/FVC 0.4), high resolution computed tomography (HRCT) typical findings, absence of lung infections at bronchoalveolar lavage (BAL) and the concomitant presence of distinctive manifestations of cutaneous and ocular cGVHD [11]. Over the following years, pulmonary cGVHD steadily progressed, requiring continuous administration of immune-suppressive drugs (i.e., systemic and inhaled steroids, mycophenolate mofetil or rapamycin) and, occasionally, oxygen therapy. At the end of December 2020, while the patient was under prednisone (20 mg/day) plus rapamycin (0.5 mg/twice daily), and antiviral (acyclovir 400 mg/day), antifungal (fluconazole 200 mg/day) and anti-*Pneumocystis jirovecii* (trimethoprim 80 mg and sulfamethoxazole 400 mg thrice weekly) prophylaxis, respiratory symptoms suddenly worsened. A chest HRCT showed some excavated nodular lesions, with the largest being located at the right hilum (13 × 12 mm) and left lower lobe (14 × 13 mm). Most of the lesions were contiguous with bronchial walls, others were communicating with (Figure 1A). Further HRCT findings were cGVHD-related ground glass opacities at both lower and upper lobes, and bronchiectasis at lower lobes (Figure 1B). Based on a transient positive serum galactomannan testing, a treatment with posaconazole (300 mg/day PO) was begun. However, over the following weeks rare skin lesions were appearing on patient legs and arms. At the beginning of February 2021, the patient was admitted to our hospital due to fever, worsened respiratory symptoms with continuous oxygen therapy requirement, and an increased number of painful, erythematous, swollen skin nodular lesions. The performance status was poor (ECOG score 4) [12]. Blood tests were showing grade-3 lymphocytopenia (0.39 10^9^/L), increased C-reactive protein (CRP) levels (136 mg/L), but normal procalcitonin. Sputum cultures and CMV viremia were persistently negative. At that time a central vein catheter (CVC) had to be inserted due to the lack of peripheral vein accesses. Oral posaconazole was then substituted with amphotericin B liposomal (5 mg/Kg/day IV) while the immune-suppressive therapy was continued. Considering fever persistence and increasing CRP levels, a large spectrum antibiotic therapy including piperacillin/tazobactam and linezolid IV was prescribed in the following days. Hard and painful skin nodules were now scattered all over the body, rapidly increasing in size, with some of them causing abscesses (Figure 2A,B). Opioids were needed to control pain. Daily dressings were required. To establish the nature of such lesions, a biopsy of a nodule of the right thigh was done. The histological analysis documented a diffuse nodular suppurative inflammation involving dermis and subcutis (Figure 2C), while the Ziehl-Neelsen staining revealed numerous acid-fast bacilli (Figure 2D). After 88 h incubation (BD Bactec FX40 blood culture system) concomitant blood cultures from both CVC and peripheral veins turned out positive for acid-fast bacteria that were further characterized as *Mab* subsp. *abscessus* by molecular analysis (DNA-STRIP technology, GenoType Mycobacterium CM). To establish the infection extent the patient underwent a CT/PET showing an increased FDG-uptake at skin and soft tissues levels, with those of the right tight and sacrum presenting the maximum value (SUV 5.4) (Figure 2E). Lung lesions were also positive (Figure 2F). MRI ruled out any brain or eyes involvement. Due to the patient poor clinical conditions, the availability of microbiological evidence from blood culture and skin biopsy, and a radiological/nuclear lung imaging consistent with a *Mab*-related disease, BAL was not performed. Based on the diagnosis of disseminated *Mab* infection, in the second half of February 2021 the patient began an empiric multi-drug antibiotic treatment including amikacin (500 mg/day IV), azithromycin (500 mg/day PO), imipenem (500 mg/three times daily IV), clofazimine (100 mg/day PO), linezolid (600 mg/twice daily IV), and tigecycline (50 mg every 12 h IV, 100 mg loading dose), the latter being stopped 48 h later due to adverse reaction. Worthy of note, a few days later the antibiotic susceptibility testing performed on *Mab* isolates from blood culture was confirming the sensitivity to amikacin and azithromycin (Broth microdilution, CLSI M62, 1st edition, November 2018). Following a 1-month multi-drug antibiotic treatment, patient respiratory symptoms were slightly improving, and the oxygen therapy requirement reduced. A pulmonary rehabilitation program could be initiated even tough a chest HRCT performed in the same days was showing the persistence of excavated nodular lesions and ground glass opacities at both the lower and upper lobes. At clinical examination skin lesions were reduced in number but still present all over the body, either in nodular or abscessed form. After 2 months of multi-drug antibiotic therapy, based on respiratory symptoms improvement and reduction of CRP level, the patient was discharged from the hospital with an at home care program including oxygen therapy, pulmonary rehabilitation, and the administration of amikacin (750 mg/three times weekly IV), azithromycin and clofazimine PO, together with the usual immune-suppressive treatment for pulmonary cGVHD. Weekly hospital visits were also scheduled. At the end of April 2021, following a 2.5 months treatment, skin lesions began to worsen, especially on legs, and linezolid (600 mg/day PO) was reintroduced to potentiate the ongoing antibiotic therapy. In the same days a new chest HRCT was showing no improvement. At that time, azithromycin was stopped due to the previous identification, at drug susceptibility testing (14 day incubation), of the inducible macrolide resistance *erm*(41) gene (DNA-STRIP technology, Genotype NTM-DR). In the following weeks, while in multi-drug antibiotic treatment, the patient experienced a further worsening of respiratory function. Finally, at the end of June 2021, 4.5 months after the diagnosis of disseminated *Mab* infection, the patient died of heart failure while in a severe status of cachexia. At that time skin lesions were still present. According to serial blood cultures performed throughout the whole antibiotic treatment, and despite CVC substitutions, *Mab* clearance was never obtained.

## 3. Discussion and Literature Review

Environmental NTM are increasingly recognized as a source of pulmonary infection in patients affected by underlying lung disease or with impaired immunity [1,2,3]. Consistently, NTM have been described as a possible cause of lung disease even in allogeneic HSCT settings, with patients affected by cGVHD and CMV viremia being at highest risk [5,6]. Despite regional variability, *Mab* is quite rare as compared to other NTM species. For instance, the most common species isolated in a cohort of twenty alloHSCT Canadian patients with NTM pulmonary disease was *Mycobacterium avium,* accounting for 50% of cases [13]. However, a single-center report from Japan has recently described three cases of macrolide-resistant *Mab*-related lung infections in as many alloHSCT patients previously treated with macrolides for bronchiolitis obliterans, a pulmonary manifestation of cGVHD [14]. Table 1 resumes subspecies, characteristics and outcome of *Mab* infection in adult alloHSCT patients according to the literature published in the last 10 years [13,14,15,16,17,18,19].

Our patient developed a disseminated *Mab* subsp. *abscessus* infection with lung, skin, and soft tissues involvement and hematogenous spread. Obviously, both cGVHD-related obstructive and restrictive pulmonary changes and the prolonged immune-suppressive therapy required to control cGVHD, favored lung disease and disseminated infection. Further predisposing factors for *Mab* infection were the patient low body mass index and the prolonged use of inhaled steroids, while no macrolides were used since the diagnosis of pulmonary cGVHD [3]. Numerous environmental *Mab* sources including soil, water system, and contaminated materials have been described in literature, while the patient-to-patient spread is debated [8,10]. Susceptible hosts can be infected through inhalation, wounds, or catheters. Hospital outbreaks were also described [10]. At the infection onset our patient had no CVC inserted or wounds and used to stay at home in self-isolation due to COVID-19 pandemic. Nonetheless, she was exposed to potential *Mab* sources as she used to live in an ancient building with damp wall issues, and to garden domestic plants. Moreover she was using oxygen devices. Unfortunately, due to the absence of previous NTM infections among our patients, and in agreement with the European Group for Bone Marrow Transplant advice [20], we did not perform a regular screening for lung NTM colonization or infection. Nonetheless, the diagnosis of NTM infection by microbiological analysis of sputum samples is challenging as specimen collection and testing may need to be repeated on several occasions [1]. In addition, *Mab* isolates from sputum do not always reflect the diversity of subclones with different antimicrobic resistance profiles [7]. A few NTM are regarded as pathogens for humans, however they are ubiquitous in the environment [4]. Our patient was not adequately warned of the potential environmental sources of *Mab*, however it seems quite difficult to envision effective preventive measures in daily life. Moreover, despite the significant global impact of opportunistic NTM, there are no vaccines currently available [21]. Although a prompt removal of CVC is required in case of rapidly growing mycobacterial bacteremia [22], the need of continuous IV infusions and the lack of peripheral vein accesses did not allow us avoiding this device during the whole antibiotic treatment. Therefore, despite CVC substitutions, our patient never achieved a microbiological blood clearance.

According to official guidelines pulmonary *Mab* infections require a combination of three or more active drugs that must be selected by drug susceptibility testing among macrolides, amikacin, imipenem, cefoxitin, tigecycline, linezolid, and clofazimine [23]. Despite such combination regimens pulmonary infections are associated to a high rate of treatment failure and poor clinical prognosis [9,22,23,24]. Currently, there is no consensus on the optimum duration of the multi-drug therapy [23]. With regard to disseminated infection, the evidence on treatment is extremely scarce and no standard antibiotic regimens have been identified outside those already adopted for lung involvement. A major problem encountered in the treatment of *Mab* infections is the innate or acquired resistance to macrolides, the cornerstone of *Mab* multi-drug treatment. Innate resistance mostly relies on a inducible erythromycin methylase *erm*(41) gene [7,9] that is functional in subsp *abscessus* and subsp *bolletii*, but not in subsp *massiliense*. [7]. Importantly, as observed in the case of our patient, in vitro drug susceptibility testing may turn out negative for macrolide resistance at day 3, showing *erm*(41) induction only upon a longer (i.e., 14 day incubation) drug exposure [7,25]. Acquired resistance mechanisms to macrolides may involve the gene encoding a 23S peptidyl transferase in the large 23S ribosomal subunit (*rrl)*, and a T to C mutation at position 28 on *erm*(41) [7,25].

## 4. Conclusions

Due to the lack of registry-based studies the real impact of *Mab*-related infections in alloHSCT setting is currently unknown. Nonetheless, our experience and data from literature indicate that in alloHSCT and related complications *Mab* may cause severe and life-threatening infections, either lung-restricted or disseminated. While patients should be warned of the potential environmental sources of NTM, microbiological surveillance is limited to pulmonary disease, even though optimal screening methods have not been clearly identified yet. A close collaboration with expert microbiologists and infectious diseases consultants is crucial to obtain a prompt diagnosis and tempt a proper treatment. However, only the development of research on vaccines and new drugs will provide an effective chance of prevention and cure for *Mab* infections in alloHSCT patients [9,21].

## Figures and Tables

**Table 1:** Subspecies, characteristics and outcome of *Mab* infection in adult alloHSCT patients according to the literature published in the last 10 years.

Ref.	Sex/Age	Subsp.	Sites Involved	Time from alloHSCT (Months)	Immune Suppressive Therapy	GVHD	First-Line Therapy	Response	Infection Outcome/Status
Miyake et al. [14]	27/M	*Abscess.*	Lung	51	PDN, CNI, IM	cGVHD lung	CAM, AMK, MPM, STFX	Recurrence	Second-line therapy/Alive
	47/M	*Abscess.*	Lung	13	PDN, CNI	cGVHD lung, skin	CAM, AMK, MPM, STFX	Complete	Cured/Alive
	48/M	*Massil.*	Lung	63	PDN, CNI, InS	cGVHD lung	CAM, AMK, MPM, STFX	Complete	Cured/Alive
Salvator et al. [15]	72/F	NA	Lung	48	PDN, CNI, ECP, RUX	cGVHD skin	AZM, AMK, IPM	Complete	Cured/Alive
Hirama et al. [13]	36/F	NA	Lung	11.8	PDN MMF	cGVHD lung and liver	AZM, MXF, AMK, FOX, LZD	Recurrence	NA/Unrelated death
	45/F	NA	Lung	24	PDN MMF	cGVHD skin, eye, intestine, mouth	AZM, AMK, IPM	Complete	Cured/Alive
Xie et al. [16]	59/F	NA	Bloodstream, CVC-tunnel	1	NA	no	CAM AMK, FOX, IPM	Recurrence	Second-line therapy/Unrelated death
Musharbas et al. [17]	69/M	*Massil.*	Bloodstream, lung, skin	NA	NA	no	CAM IPM, AMK, TIG,	Progression	Progression/Related death
Toyama et al. [18]	51/M	*Massil.*	Bloodstream, lung, soft tissue	0	NA	no	ETAM, CAM	Progression	Cured/Alive
Nagata et al. [19]	62/F	NA	Bloodstream, muscle, joints	19	PDNS	no	CAM, IPM, AMK, MINO	Recurrence	Second-line therapy/Unrelated death
	67/M	NA	Bloodstream, CVC-related	0	NA	no	CAM, IPM, AMK	Complete	Cured/Alive
Present	34/F	*Abscess.*	Bloodstream, lung, skin, soft tissue	56	PDN, SLM, InS	cGVHD lung (Skin and eye)	AZM, CFZ, AMK, LZD, IPM	Progression	Progression/Related death

Ref.: Reference; Subsp: Subspecies; alloHSCT: Allogeneic Hematopoietic Stem Cell Transplant; GVHD: Graft versus Host Disease; cGVHD: Chronic GVHD; M: Male; F: Female; NA: Not Available; CVC: Central Vein Catheter. PND: Prednisone; CNI: Calcineurin Inhibitor; IM: Imatinib; InS: Inhaled Steroid; ECP: Extracorporeal Photopheresis; RUX: Ruxolitinib; MMF: Mycophenolate Mofetil; PDNS: Prednisolone; SLM: Sirolimus.; CAM: Clarithromycin; AMK: Amikacin; MPM: Meropenem; STFX: Sitafloxacin; IPM: Imipenem; AZM: Azithromycin; MXF: Moxifloxacin; FOX: Cefoxitin; LZD: Linezolid; FOX: Cefoxitin; TIG; Tigecycline; ETAM: Ethambutol; MINO: Minocycline; CFZ: Clofazimine.

## Data Availability

Not applicable.
